# Spina bifida as a multifactorial birth defect: Risk factors and genetic underpinnings

**DOI:** 10.1002/pdi3.2517

**Published:** 2025-01-25

**Authors:** Ethan S. Wong, Daniel A. Hu, Lily Zhang, Rachel Qi, Cindy Xu, Ou Mei, Guowei Shen, Wulin You, Changqi Luo, Tong‐Chuan He, Russell R. Reid, Lewis S. Shi, Michael J. Lee, Yi Zhu

**Affiliations:** ^1^ Molecular Oncology Laboratory Department of Orthopaedic Surgery and Rehabilitation Medicine The University of Chicago Medical Center Chicago IL USA; ^2^ Pritzker School of Medicine The University of Chicago Medicine Chicago IL USA; ^3^ Department of Orthopedic Surgery Jiangxi Hospital of Traditional Chinese Medicine Jiangxi University of Traditional Chinese Medicine Nanchang China; ^4^ Department of Orthopaedic Surgery BenQ Medical Center The Affiliated BenQ Hospital of Nanjing Medical University Nanjing China; ^5^ Department of Orthopaedic Surgery Wuxi Hospital Affiliated to Nanjing University of Chinese Medicine Wuxi China; ^6^ Department of Orthopaedic Surgery Yibin Second People’s Hospital Affiliated with West China School of Medicine Yibin China; ^7^ Laboratory of Craniofacial Biology and Development Section of Plastic and Reconstructive Surgery Department of Surgery The University of Chicago Medical Center Chicago IL USA

**Keywords:** genetics, neural tube defects, risk factors, spina bifida, spinal dysraphism

## Abstract

Spina bifida is a birth defect resulting from abnormal embryonic development of the neural tube. Though spina bifida is divided into several subtypes, myelomeningocele—the most severe form of spina bifida often associated with a markedly diminished quality of life—accounts for a significant portion of cases. A broad range of genetic and environmental factors, many of which are still unknown, influence spina bifida, making it difficult to provide a comprehensive etiology for the disorder. Folic acid supplementation aided by the mandatory fortification of food is preventive; still, spina bifida persists due to numerous other confounding factors that affect risk. This article reviews the latest studies pertaining to the risk factors and genetics involved in spina bifida in an attempt to elucidate the complex background of the congenital malformation. Additionally, this review highlights the significant impact of environmental pollutants, adverse medication effects, and maternal health conditions such as diabetes and obesity on the prevalence of spina bifida. Emerging research on gene‐environment interactions provides insight into how specific genetic variants may influence susceptibility to these environmental factors. We also discuss new technologies in genetic sequencing that show promise for the large‐scale discovery of genes associated with spina bifida risk. Understanding these intricate interactions is crucial for developing effective prevention and intervention strategies.

## 1 INTRODUCTION

Spina bifida, also called spinal dysraphism, is a congenital malformation of the central nervous system (CNS). The anomaly is associated with a wide range of conditions and debilitating deficits including hydrocephalus, Chiari II malformation, paralysis, and neurogenic bladder and bowel.^1^ It is one of the most common congenital defects, affecting an estimated one in every 2875 children in the United States and carrying a 4.4% infant mortality rate.^2^, ^3^ Spina bifida falls under an umbrella group of CNS malformations called neural tube defects (NTDs), which includes other defects such as anencephaly and encephalocele.

**FIGURE 1:**
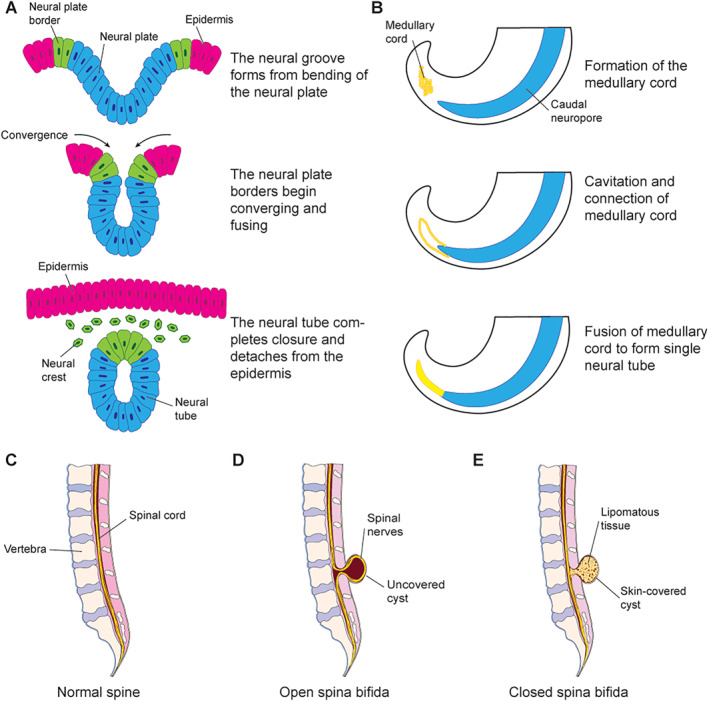
Neurulation and its relation to the development of spina bifida subtypes. (A) Schematic representation of transverse sections showing the normal progression of primary neurulation. (B) Schematic representation of sagittal sections showing the normal progression of secondary neurulation. (C) Schematic representation of a sagittal section showing normal spinal anatomy. (D) Schematic representation of a sagittal section showing the anatomy of an open spina bifida defect, specifically myelomeningocele, caused by failure during primary neurulation. (E) Schematic representation of a sagittal section showing the anatomy of a closed spina bifida defect, specifically lipomeningocele, caused by failure during secondary neurulation.

Spina bifida is a broad term that can be more accurately classified into divisions and subdivisions depending on factors such as vertebral level, severity, and the presence of a layer of skin covering the defect. Meaning “split spine” in Latin, spina bifida fundamentally refers to all congenital spinal disorders involving disruption in the midline closure or differentiation of mesenchymal, osseous, and neural tissue during neurulation. Neurulation is a complex process that develops the neural tube—the precursor of the brain and spinal cord—and begins in the third week of gestation. Neurulation is separated into a primary phase that forms the cranial portion of the neural tube and a secondary phase that forms the caudal portion. A disruption during primary neurulation is typically associated with spina bifida aperta—also known as open spina bifida, spina bifida cystica, or open spinal dysraphism—which includes myelomeningocele. Myelomeningocele, considered the most common and severe form of spina bifida, is estimated to comprise 85% of all spina bifida cases.^4^, ^5^ In contrast, a disruption during secondary neurulation is associated with most closed‐type spina bifida cases such as spina bifida occulta and lipomeningocele. Anencephaly is a much more severe NTD that occurs early in neurulation due to the neural tube failing to close at the rostral end. These alterations during embryological development have a direct relationship to the specific malformation that they lead to, with the more severe defects occurring at earlier stages of gestation (Figure 1).^6^, ^7^

The specific causes that give rise to these alterations of the neural tube are multifaceted and cannot be explained by a single gene or risk factor. Rather, a plethora of genetic and environmental factors interact to influence the genesis of the malformation. While decades of research have helped provide a clearer understanding of the etiology of spina bifida, the full extent of genes and risk factors that play a role in this disorder is still unknown. Important discoveries such as the significant risk mitigated by folic acid supplementation have helped decrease the prevalence of spina bifida, but cases that are not folate‐sensitive or involve other confounding factors remain. In this review, we introduce the current understanding of the risk factors and genetics underlying spina bifida. We then summarize the technological advances thus far that have allowed for further elucidation of the implicated gene pathways in spina bifida development.

## 2 MATERIALS AND METHODS

### 2.1 Literature search

**FIGURE 2:**
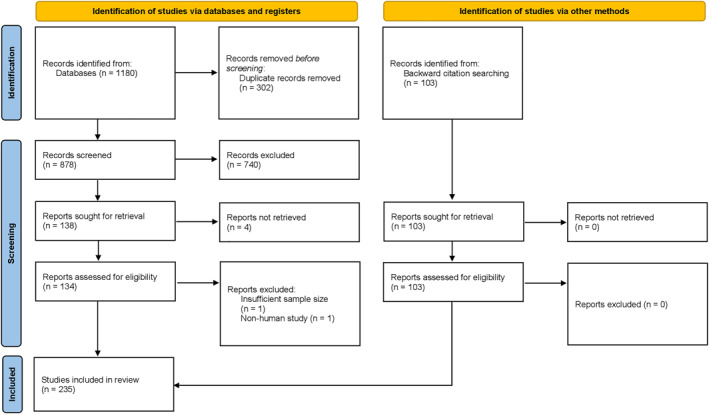
PRISMA flow diagram. A comprehensive literature search was conducted using Boolean operators across the PubMed, Embase, and Cochrane Library databases in accordance with the Preferred Reporting Items for Systematic reviews and Meta‐Analyses extension for Scoping Reviews (PRISMA‐ScR).^8^

A comprehensive literature search was conducted using Boolean operators across the PubMed, Embase, and Cochrane Library databases in accordance with the Preferred Reporting Items for Systematic reviews and Meta‐Analyses extension for Scoping Reviews (PRISMA‐ScR).^8^ The database search was limited to full‐text human studies in the English language published from 2003 through 2023 to find the most relevant information. Search terms included and related to “spina bifida”, “genetics”, and “risk factors”, according to the National Library of Medicine Medical Subject Headings (NLM MeSH). Across all database searches, terms for “spina bifida” were exclusively searched in the title to only identify articles with a primary focus on spina bifida rather than other conditions. All other search terms were searched in both the title and abstract. The full search strategy for each database is available in Supplement Table 1. Additional studies were identified through a backward citation search of the references from articles selected for inclusion following full‐text review (Figure 2). Studies found through citation searching were not subject to any of the restrictions placed on search results from the databases.

### 2.2 Eligibility criteria and screening

All eligibility criteria were established a priori. To be eligible for inclusion, studies must have investigated the risk factors or genetics of spina bifida. Non‐human studies, book chapters, abstracts, letters, comments, case reports, studies not in English, and studies with insufficient spina bifida‐specific sample sizes (*n* < 5) were excluded. After removing duplicates, all titles and abstracts were independently screened for eligibility by four investigators using the Rayyan screening tool.^9^ Full‐text articles were then randomly divided among investigators and retrieved to further assess the remaining studies against the selection criteria, except articles where full‐text could not be found. Disagreements between the investigators were resolved through discussion and consensus with the help of a fifth investigator. Due to substantial differences in study design, methodology, and outcomes, a critical appraisal of individual sources of evidence was not conducted.

## 3 RISK FACTORS

Spina bifida is considered to have a complex, multifactorial origin arising from both environmental and genetic factors that interact to form the congenital disorder.^10^ A small proportion of spina bifida cases occur in association with a defined chromosomal, teratogenic, or Mendelian malformation syndrome.^11^, ^12^, ^13^, ^14^, ^15^, ^16^, ^17^, ^18^, ^19^ It is estimated that risk factors account for approximately 28% of all spina bifida risk, which is consistent with other estimates that the heritability of spina bifida is approximately 70%.^10^, ^20^ The risk factors for spina bifida are difficult to definitively quantify; however, there are select factors that have been well‐established and shown to be especially significant in the genesis of the condition. A summary of risk factors is presented in Table 1.

**TABLE 1:** Risk factors implicated in spina bifida.

Risk factors	References
Folate supplementation	
Inadequate folic acid supplementation	24, 25, 26
Environmental exposure	
Maternal exposure to solvents and pesticides	24
Maternal exposure to selenium	45
Maternal exposure to methylmercury	51, 52, 53
Maternal exposure to agricultural chemicals	54, 55
Maternal exposure to chlorinated solvents	56
Maternal exposure to 50 Hz electromagnetic fields	57
Paternal exposure to agent orange	41, 44
Paternal exposure to arsenic	45
Paternal exposure to organic solvents	43
Maternal occupational exposure to anesthetic gas	58
Paternal occupational exposure to welding fumes	59
Paternal occupational exposure to UV radiation	59
Medications	
Valproic acid	32, 61
General anticonvulsant monotherapy	25, 41
Folic‐acid antagonists	63
Antimalarials	73
Guaifenesin	74
Nitrosatable drugs	75, 76
Antipyretics	24, 77
Infertility medications	106
Maternal diabetes and weight	
Diabetes mellitus	79, 80, 81, 82
Obesity (BMI ≥ 30)	39, 40, 41, 87, 90, 91, 92, 93, 94
Overweight (BMI 25–29.9)	77
Interpregnancy increase in BMI	95
Insufficient gestational weight gain	77
Family and obstetric history	
Family history of spina bifida	25, 77, 78, 96, 97, 98, 99, 100, 101, 102, 103
Infertility	106
History of abortion	94
History of stillbirth	107
Multiple birth	101, 108
Birth order: second born	24
Birth order: third born and later	24
Other maternal risk factors	
Maternal fever	41, 77, 78, 110, 111, 112, 113, 114, 115
Cytomegalovirus	117
Common cold	77
Asthma	118
Low vitamin B12	119, 120
Smoking	24
Caffeine intake	24, 78, 121, 122

Abbreviation: UV, ultraviolet.

### 3.1 Inadequate folate supplementation

One of the most well‐established risk factors for spina bifida is inadequate maternal consumption of folate. Folate, which can also be derived through its synthetic form of folic acid, is essential to many critical processes involved in fetal development. Thus, a lack of the B vitamin can lead to NTDs. Additionally, folate deficiency can lead to elevated homocysteine, which is believed to be directly teratogenic. Specific genetic mutations within the folate‐homocysteine pathway may worsen this existing relationship between spina bifida, folate, and homocysteine by affecting the metabolism of folic acid.

Spina bifida is considered to be generally preventable through periconceptional folic acid supplementation at a dose of 0.4 mg each day, which has shown to reduce the first‐time risk and recurrence risk of NTDs by about 70%.^21^, ^22^, ^23^ The majority of studies investigating the use of supplementation found lack of supplementation to be a significant risk factor with varying degrees of increased risk. One study directly investigating plasma folate levels found that lower maternal plasma folate levels led to more than eightfold greater risk than higher levels.^11^ ^,^ ^24^, ^25^, ^26^, ^27^ A landmark prevention trial by the MRC Vitamin Study Research Group in 1991 showed this reduction in risk, which significantly contributed to the push in supplementation guidelines and later folic acid fortification of food.^21^

Due to the large proportion of spina bifida being folic acid‐preventable, many countries enacted mandatory nutrition programs in an effort to mitigate these preventable defects.^28^ This mandatory fortification is associated with a considerable increase in folate status.^25^, ^29^, ^30^, ^31^ Furthermore, when comparing the case severity of spina bifida before and after mandatory folic acid fortification in the United States, a reduction in prevalence by 19%–34% was found.^32^, ^33^, ^34^, ^35^ However, despite the apparent success of folic acid in preventing spina bifida, lipomyelomeningocele—a closed subtype of spina bifida characterized by a cyst containing lipomatous tissue—is seemingly unaffected by fortification. In the full fortification period, the prevalence of lipomyelomeningocele did not significantly change.^36^, ^37^ In fact, in one population‐based study, the rate of lipomyelomeningocele even began a trend of increasing occurrence, although, the increase was not statistically significant.^36^, ^38^ Folic acid supplementation, though, has been shown to reduce the risk of lipomyelomeningocele.^39^ This suggests that lipomyelomeningocele is distinct from other subtypes of spina bifida, namely myelomeningocele.

While folic acid supplementation alone can significantly reduce risk, it may only attenuate and not eliminate the additional risk posed when combined with other risk factors. Furthermore, the odds of spina bifida often become more significant when lack of folic acid is combined with another risk factor. For example, it was found that the risk of spina bifida from obesity was affected little by supplementation, suggesting that some risk factors may outweigh the benefits of folic acid.^40^, ^41^, ^42^

### 3.2 Exposure to chemicals and environmental pollutants

A wide range of pollutants and substances are known to have particularly negative effects on health and can be extremely dangerous to a developing fetus. Organic solvents have been indicated to influence fetal development from both a paternal and maternal exposure standpoint. They dissolve organic substances such as lipids, have widespread use, and are agents rampant in the painting, dry‐cleaning, printing, and chemical industries. Paternal exposure to organic solvents was found to have increased odds of having a child with NTDs (OR = 1.86, 95% CI 1.40–2.46) while another study found over tenfold increased odds from maternal solvent and pesticide exposure (OR = 10.62, 95% CI 1.23–91).^24^, ^41^, ^43^ Agent Orange, composed of 2,4,5‐T and 2,4‐D and traces of toxic dioxin, is a specific organic solvent that was used by the United States in the Vietnam War and was suspended from use following evidence of major negative health effects. A study found that paternal Agent Orange exposure doubled the risk of having a child with spina bifida (RR = 2.02, 95% CI 1.48–2.74).^41^, ^44^ This suggests that certain organic solvents may contribute to spina bifida risk more than others and should each be individually investigated to understand their implications on fetal health.

Additionally, many elemental metals have been shown to be associated with a higher risk of giving birth to a child with spina bifida. Arsenic, a well‐established environmental pollutant that naturally occurs in soil, can be inhaled or ingested through contaminated food and water. It is a carcinogen that is associated with skin lesions, cardiovascular disease, impaired neurological function, diabetes, and several types of cancer.^45^, ^46^, ^47^ Arsenic has been suggested to indirectly increase the risk of spina bifida through the depletion of necessary methyl groups for cell division and tissue growth and that chronic arsenic exposure may lead to diabetes, which is a risk factor in itself.^48^, ^49^ To this end, paternal exposure to arsenic bears an increased odds ratio in the development of spina bifida in one study (OR = 1.74, 95% CI 1.26–2.42). This study also investigated exposure to other metals and found that paternal exposure to aluminum, cobalt, chromium, iron, selenium, and vanadium were all associated with increased odds of spina bifida. For maternal exposure, selenium was associated with increased odds (OR = 4.82, 95% CI 1.32–17.60) while zinc exposure was associated with lower odds (OR = 0.11, 95% CI 0.03–0.42).^45^ Zinc is an important antioxidant and thus in high concentrations may aid in the prevention of oxidative stress and, consequently, oxidative damage, which may explain its protective role in spina bifida development.^50^ The gender‐specific exposure risks are interesting as they may indicate differential contribution of X‐ or Y‐chromosome specific loci important in neurulation.

Methylmercury, an extremely potent neurotoxin that readily passes through the placenta, has also been shown to increase the risk of spina bifida with exposure through a dose‐response relationship.^51^, ^52^, ^53^ Maternal exposure to other occupational chemicals, chlorinated solvents, agricultural pesticides, electromagnetic fields, and anesthetic gases were also found to increase the risk of an offspring with spina bifida.^41^, ^54^, ^55^, ^56^, ^57^, ^58^ Additionally, paternal exposure to welding fumes and ultraviolet (UV) radiation during welding were associated with higher odds of spina bifida.^59^ However, some of these associations are not as well‐established and require further investigation.

The risk posed by these environmental pollutants is suggested to be related to gene‐environment interactions. For example, specific polymorphisms of genes within the folate‐homocysteine pathway may increase the risk that arsenic poses in the development of spina bifida.^48^, ^49^ Other interactions may interfere with the body’s ability to break down toxic chemicals as well.^60^ These polymorphisms could further augment the pre‐existing effect of specific environmental pollutants or independently lead to an increased risk of spina bifida.

### 3.3 Adverse effects of medications

In terms of pharmacological risks, numerous medications, specifically anticonvulsants, are known teratogens with varying consequences including NTDs. Valproic acid and carbamazepine in particular are older generation anticonvulsants associated with increased risk of spina bifida; valproic acid alone has been shown to increase risk by at least tenfold, proving the drug to be very potent in the context of spina bifida.^25^, ^32^, ^41^, ^61^ Interestingly, the valproate moiety has been well established as a teratogen in other diagnoses, such as cleft lip^62^; the common denominator here is that valproate must inhibit pathways key for the fusion of tissues. Valproic acid and carbamazepine are also known folic acid antagonists, which may be the mechanism through which they increase the risk of spina bifida. Due to inadequate folate being a well‐established risk factor for spina bifida, the association with folic acid antagonists is clear. Other folic acid antagonists with known associations to spina bifida include aminopterin, methotrexate, and trimethoprim. Folic‐acid antagonist exposure in general was associated with a nearly threefold increase in odds (OR = 2.8, 95% CI 1.7–4.6), with exposure specifically to carbamazepine and trimethoprim being even higher.^11^, ^63^ Women who are prescribed anticonvulsants are recommended to take a higher daily dose of 5 mg of folic acid as opposed to the typical recommended periconceptional dose of 0.4 mg.^64^ Additionally, folic acid supplementation in tandem with either carbamazepine or trimethoprim significantly reduced the risk presented by the drugs alone.^63^ This further suggests that the specific mechanism under which many of these teratogens operate relates to folic acid.

Unlike enzyme‐inducing anticonvulsants, it has been suggested that folic acid does not have much of an effect on the risk posed by valproic acid as the drug does not reduce total serum folate levels.^41^, ^64^, ^65^, ^66^ Though folic acid supplementation lowers the risk of spina bifida from anticonvulsants such as carbamazepine, it still does not completely remove risk, even from enzyme‐inducing anticonvulsants. This indicates that anticonvulsants may operate under a different mechanism than other folic acid antagonists or may have a second mechanism that further augments the risk of spina bifida: free radical‐mediated cellular damage during neurodevelopment.^11^, ^67^ Typically, antioxidants and other scavenging enzymes work to remove free radicals; however, the metabolism of anticonvulsants creates an increased burden of free radicals and may also interfere with already overloaded scavenging activity, creating a state of oxidative stress.^67^, ^68^, ^69^, ^70^ This allows for significant cellular damage during embryonic development, which can lead to spina bifida.^68^, ^70^, ^71^ Phenytoin, carbamazepine, valproic acid, phenobarbital, and benzodiazepine monotherapy and polytherapy all resulted in elevated concentrations of the oxidative damage marker lipid peroxide, signifying that oxidative stress is likely one of the mechanisms through which anticonvulsants increase spina bifida risk.^67^ Though there is data showing folic acid supplementation leading to a reduction of risk in carbamazepine and other teratogens,^63^ additional investigation is needed to determine whether higher doses may do the same for valproic acid. Newer generation antiepileptic drugs may be preferred during gestation due to their better toleration and lack of antifolate properties, but the relatively limited data on these medications warrants further exploration. There are conflicting results on whether maternal epilepsy itself influences the risk of spina bifida through restriction of circulation to the fetus during seizures; however, it is recommended that women should not stop antiepileptic medication during pregnancy, as seizures pose a higher risk for both the mother and child.^72^

Many antimalarial drugs including sulfonamides and artemisinin act through the deprivation of folate from the malaria parasite, which is essential for its survival. As such, there is a potential risk for spina bifida, with one study finding a significantly higher occurrence of spina bifida in mothers who ingested the antiparasitics.^73^ Guaifenesin, an expectorant medication used to thin mucus and phlegm, has also been shown to increase the odds of spina bifida (OR = 2.2, 95% CI 1.1–4.3).^74^ Nitrosatable drugs span various drug classes and can form *N*‐nitroso compounds, which have been shown to be carcinogenic and induce congenital abnormalities in animals, raising concern about their effect in humans. Nitrosatable drugs were found to be associated with up to nearly threefold spina bifida odds.^75^, ^76^ The intake of infertility medications such as clomiphene citrate (OR = 10.9, 95% CI 2.5–36.7) has also been shown to increase the odds of a child with spina bifida. Use of antipyretic medications has conflicting results, with studies showing it to be both a risk factor and protective factor for the malformation.^24^, ^77^, ^78^ Additionally, the inherent need for an underlying condition, makes the data on antipyretics and infertility medications as independent risk factors more nuanced and difficult to elucidate.

### 3.4 Maternal diabetes and weight

Endocrinological and metabolic factors may also contribute to the pathogenesis of NTDs. Maternal diabetes mellitus, both pregestational and gestational, predisposes a child for spina bifida and various other congenital malformations. The odds of spina bifida is increased two‐ to sixfold with maternal diabetes mellitus.^12^, ^79^, ^80^, ^81^, ^82^ The exact mechanism through which diabetes increases risk is unknown; however, it has been shown in animal studies that hyperglycemia is directly teratogenic and induces embryopathy.^11^, ^80^, ^83^, ^84^ Having pregestational diabetes carries a higher risk of having a child with any congenital malformation than gestational diabetes.^11^, ^79^ This is potentially due to pregestational diabetes being already present during major developmental stages, whereas gestational diabetes typically does not form until after the majority of congenital malformations develop.^85^ Folic acid lessens, but does not eliminate, the risk presented by diabetes.^40^, ^86^

Obesity (BMI ≥ 30) is associated with major health implications for both the mother and child. Obesity is especially high‐risk, as chronic malnutrition and higher prevalence of other conditions, including maternal diabetes, often accompany obesity and carry their own negative effects. Mothers considered to be overweight (BMI = 25–29.9), obese, and severely obese (Class III, BMI >40) have twofold, nearly fourfold, and nearly ninefold odds, respectively, of developing gestational diabetes mellitus, which further increases the risk observed by obesity alone.^12^, ^41^, ^87^, ^88^, ^89^ Without the added risk of diabetes, obesity increases the odds of spina bifida two‐ to over threefold than those of healthy weight (BMI = 18.5–24.9).^39^, ^40^, ^41^, ^87^, ^90^, ^91^, ^92^, ^93^, ^94^ The risk of spina bifida in patients with underweight status is negligible, but one study found that overweight status is associated with increased risk (OR = 2.5, 95% CI 1.6–4.2).^77^, ^92^ As mentioned previously, folic acid does not appear to have an effect, neither positive nor negative, on the association between obesity and spina bifida.^40^, ^42^

Prepartum changes in BMI was found to have a minor, but statistically significant increase in odds for each unit increase in BMI during gestation (OR = 1.05, 95% CI 1.02–1.09).^95^ Weight gain that was categorized as insufficient was also shown to carry an increased risk of spina bifida (OR = 1.9, 95% CI 1.1–3.1).^77^

### 3.5 Family and obstetric history

Having a family member with spina bifida can, depending on the degree of consanguinity, increase the risk of having a child with spina bifida. Already having a child with an NTD carries the largest risk, with a recurrence risk between 1.8% and 8%.^11^, ^96^, ^97^, ^98^, ^99^, ^100^, ^101^, ^102^ One study reported a higher twin concordance rate of 6.8% than their reported full sibling recurrence rate of 1.8%, indicating that there may be an important polygenic etiology creating a higher incidence.^11^, ^101^ Increased risk becomes less severe as degrees of consanguinity become more distant, with second‐degree relatives being 0.5%–2% and third‐degree relatives being 0.17%–0.43%.^98^, ^103^ Some studies that provided an odds ratio of spina bifida with a family history of the condition rather than a recurrence rate reported an extremely large increase in odds, ranging from 4 to 43 times the odds.^25^, ^77^, ^78^ It is worthy to note that one recent survey study found that 16.9% of children with spina bifida have a family history of NTDs, reinforcing the theory that genetics play a large role in the condition.^104^, ^105^ The vast majority of the familial studies conducted to determine whether family history is a factor in spina bifida are from the 1980s; thus, additional investigations should be undertaken with the now larger pool of cases and technological advances in genomic analysis available.

Infertility was observed to be associated with a fourfold increase in odds (OR = 4.3, 95% CI 1.01–14.0).^106^ Mothers with a history of abortion have higher odds of giving birth to a child with spina bifida (OR = 4.9, 95% CI 1.9–12.8),^94^ as well as women who have previously had a stillbirth (OR = 2.7, 95% CI 1.2–6.3).^107^ Additionally, having a multiple birth is also associated with a higher chance of a child with spina bifida (RR = 2.09, 95% CI 1.74–2.52).^108^ Birth order shows an association that strengthens from second born (OR = 2.15, 95% CI 1.25–3.69) to third and later (OR = 3.93, 95% CI 1.69–9.17).^24^ Maternal age at delivery also presents an increased risk, but the specific age ranges differ between several studies.^24^, ^78^, ^109^

### 3.6 Other maternal risk factors

There are many other risk factors that have not yet been well‐established but have shown to be associated with spina bifida by some studies. There is evidence that maternal fever, usually caused by any viral illness, is associated with increased risk in giving birth to a child with spina bifida.^11^, ^41^, ^77^, ^78^, ^110^, ^111^, ^112^, ^113^, ^114^, ^115^ However, it is unclear whether this increased risk is due to the hyperthermia itself or the underlying disorder. One study found that other causes of hyperthermia such as hot tubs (RR = 2.8, 95% CI 1.2–6.5) and hot baths (RR = 21.03, 95% CI 1.16–382.5) are associated with an increase in risk for spina bifida.^111^, ^116^ Yet, many other studies reporting an association between hyperthermia and spina bifida specify the fevers to be caused by an illness, including one study showing no increased risk for mothers who reported fever not caused by flu.^110^, ^112^, ^113^, ^114^

Cytomegalovirus (CMV) infection, a prevalent viral cause of neurological congenital abnormalities, was found to have over a fourfold increase in odds (OR = 4.55, 95% CI 1.18–17.58).^117^ The common cold, which can be caused by a number of viruses, also increases the risk of spina bifida (OR = 6.8, 95% CI 3.6–12.7).^77^ One study found asthma, a respiratory disorder affecting a large number of pregnant women, to be a significant risk factor for spina bifida (OR = 4.41, 95% CI 1.61–12.1).^41^, ^118^ Low maternal vitamin B12, which aids in homocysteine regulation, is associated with an increase in spina bifida risk.^119^, ^120^ Smoking, a risk factor for many diseases like cancer, showed a nearly twofold increase in odds (OR = 1.91, 95% CI 1.91–3.14).^24^ Caffeine intake also showed an increase in risk, but the risk from specific caffeinated beverages and medications is not certain.^24^, ^41^, ^78^, ^121^, ^122^ Other maternal lifestyle, demographic, and socioeconomic factors have also been proposed in spina bifida. However, many of these studies are standalone and require further study.

## 4 GENETIC ALTERATIONS AND PREDISPOSITIONS

The influence of genetics in the development of spina bifida is complex, as a combination of abnormalities in chromosomes, variations at multiple loci and genes, and gene‐environment interactions are known to contribute to the development of spina bifida as well as affect its recurrence patterns.^11^ It is estimated that the heritability of NTDs is 70%.^10^ The current evidence suggests that there is no singular gene disorder or variation that explains familial recurrence patterns. Instead, a multitude of gene disorders and variants are currently understood to be implicated, either through an individual influence or via crosstalk. Many such abnormalities are linked because they affect pathways or mechanistic processes important to embryological development, such as neurulation.^11^ As such, many teratogenic factors and environmental interactions are also implicated. A summary is presented in Table 2.

**TABLE 2:** Pathways and gene variants implicated in spina bifida.

Pathways and gene variants	References
Folate‐homocysteine pathway	
Methylenetetrahydrofolate reductase (MTHFR)	124
Methylenetetrahydrofolate dehydrogenase 1 (MTHFD1)	11, 125
5‐Methyltetrahydrofolate‐Homocysteine methyltransferase (MTR)	126
5‐Methyltetrahydrofolate‐Homocysteine methyltransferase reductase (MTRR)	127
Wnt signaling pathway	
Cadherin EGF LAG seven‐pass G‐type receptor 1 (CELSR1)	136, 137
Prickle planar cell polarity protein 2 (*PRICKLE2*)	138
Scribble planar cell polarity protein (SCRIB)	136, 139
Oxidative stress mechanism	
Superoxide dismutase 1 (SOD1)	140
Superoxide dismutase 2 (SOD2)	140
Carbonic anhydrase	70
Erythrocyte FRSE included catalase (CAT)	70
Glutathione peroxidase (GPX)	70
Glutathione‐S‐470 transferase (GST)	70

Abbreviation: FRSE, free radical scavenging enzymes.

### 4.1 Genetic variants in the folate‐homocysteine pathway

The folate‐homocysteine pathway plays a critical role in neural tube development, and many disruptions to this pathway are associated with an increased risk of NTDs such as spina bifida. Folate is essential for DNA synthesis, repair, and methylation. After being metabolized in the body, folate forms tetrahydrofolate (THF), which is involved in one‐carbon transfer reactions essential for the synthesis of nucleotides and amino acids. As such, folate is crucial for cell division and proper fetal development in the context of NTDs. It has been well‐established that folic acid has a protective effect, and that periconceptional folic acid supplementation greatly reduces the risk of NTDs by approximately 70%.^11^, ^123^ Genes associated with the transportation and metabolism of folic acid have been investigated heavily, and the literature suggests that many variants of these genes are significantly associated with an increased risk of developing spina bifida. One prominent gene in folate and homocysteine metabolism is methylenetetrahydrofolate reductase (MTHFR) which encodes a catalyst for a key step in the metabolic process. Maternal MTHFR 677T is reported to be a risk factor.^124^ Other variants of genes in this pathway known to be risk factors include *MTHFD1*,^11^, ^125^ MTR,^126^ and MTRR (specifically MTRR 66G).^127^ It has also been found that maternal combinations of these variants carry a significant increase in the risk of spina bifida compared to individual variants.^12^, ^128^, ^129^ More recent studies employing whole‐exome sequencing (WES) have linked numerous gene variants in the folate and one‐carbon metabolism pathway to increased risk. In one such study, 45 of 837 candidate genes investigated yielded statistically significant risk increases for NTDs.^130^

### 4.2 Spina bifida‐associated nucleotide polymorphisms affected by environmental and chemical factors

There have been several gene‐environment interactions documented in the literature, ranging from differential susceptibility to environmental pollutants to specific interactions with enzymes and other substances commonly found in pesticides and industrial chemicals (Table 3). One such gene‐environment interaction involves the Paraoxonase 1 (*PON1*) enzyme, which plays a critical role in detoxifying organophosphate pesticides. Variations in the *PON1* gene can influence an individual’s ability to metabolize these pesticides by altering the catalytic activity of the enzyme, potentially increasing the risk of spina bifida in offspring exposed to these chemicals during pregnancy. Specific *PON1* polymorphisms determined to be associated with increased risk for having a child with spina bifida include: *C108T*, *L55M*, and *Q192R*.^60^

**TABLE 3:** Gene‐environment interactions implicated in spina bifida.

Environmental hazard	Interaction with	References
Paraoxonase 1 (*PON1*)		60
Arsenic exposure	*MTHFD1, SLC19A1, DNMT3A, AS3MT*	48, 49
PM_2.5_	*SLC, CYP*	131
PM_10_	*CYP1A2* ^a^	131
Maternal smoking status	*NAT1*	131, 133

^a^ Effect observed only in cases without periconceptional maternal folic acid supplementation.

As mentioned previously, arsenic is another common environmental hazard and is known to cause NTDs in animal studies. It has been proposed that specific polymorphisms in genes involved in the folate metabolism and transport pathways result in increased susceptibility to arsenic exposure in terms of developing NTDs. One case‐control study assessed 14 polymorphisms in folate metabolism pathway‐implicated genes and found that the level of inorganic arsenic in drinking water was associated with an increased risk of myelomeningocele for 4 specific polymorphisms: the AA/AG genotype of rs2236225 (*MTHFD1*), the GG genotype of rs1051266 (*SLC19A1*), the TT genotype of rs7560488 (*DNMT3A*), and the GG genotype of rs3740393 (*AS3MT*).^48^ Additionally, high levels of arsenic exposure in drinking water have also been shown to negate the protective effect of periconceptional folic acid supplementation. Another case‐control study found that an increase in drinking water inorganic arsenic concentration from 1 to 25 μg/L resulted in a change in the odds ratio of folic acid use from 0.22 to 1.03. At arsenic concentrations greater than 25 μg/L, no significant protective effect of folic acid supplementation remained.^48^, ^49^

In addition to specific substances in the environment such as inorganic arsenic, a broad range of air pollutants have been implicated. Many studies have examined interactions between maternal occupational chemical exposures and genotypes for other congenital anomalies, such as cleft palate, cleft lip, conotruncal defects, and limb deficiencies, but there is a need for further research on these interactions as they relate specifically to NTDs.^131^, ^132^ One study examined the interaction between five types of common air pollutants—nitrogen dioxide (NO_2_), nitric oxide (NO), carbon monoxide (CO), PM_10_, and PM_2.5_—and 104 gene variants independently involved in increased risk for NTDs. In summary, the interaction of PM_10_ and many *CYP*, *NAT*, and *SLC* gene variants resulted in increased risk for spina bifida. Likewise, PM_2.5_ interacted with many variants of *SLC* and *CYP* to result in increased risk. Further complicating this effect is the impact of folic acid supplementation; the study also stratified results by maternal folic acid‐containing vitamin supplementation and found that exposure to NO and the rs762551 variant of *CYP1A2* resulted in a statistically significant odds ratio of 5.2 for spina bifida in non‐vitamin users while there was no such significantly increased risk of spina bifida in vitamin users.^131^

Along with environmental exposure to air pollutants, smoking has been elucidated to have a gene‐environment effect with *NAT1*, an enzyme that acetylates aromatic and heterocyclic amines and is involved in the catabolism of folates in the folate‐homocysteine pathway. Several *NAT1* variants are independently associated with increased risk for spina bifida, and one study examined potential interactions between these variants and maternal smoking. It found that while there was no evidence of embryonic or maternal *NAT1* C1095 genotypes independently affecting spina bifida risk, the embryonic *NAT1* C1095A genotype affects the risk of spina bifida through an interaction with maternal smoking status.^131^, ^133^

In addition to *PON1*/organophosphate pesticide interactions, arsenic, air pollutants, and smoking, other environmental factors such as heavy metals, industrial chemicals, and dietary contaminants have been studied for their interactions with genetic polymorphisms in the context of spina bifida. For example, paternal exposure to welding fumes and UV radiation during welding has been associated with higher odds of spina bifida, suggesting that occupational exposures can also interact with genetic factors to influence NTD risk.^59^ Overall, the current literature suggests that certain *PON1* variants are independently associated with increased spina bifida risk through a mechanism that hinders the ability to detoxify organophosphate pesticides. Moreover, high levels of exposure to arsenic, air pollutants, and maternal smoking status each interact with other polymorphisms with various levels of independent risk to produce an overall further heightened risk for spina bifida. The understanding of gene‐environment interactions in spina bifida is crucial for developing targeted prevention strategies. Interventions could include minimizing exposure to specific environmental hazards for individuals with known genetic susceptibilities. Further research should focus on elucidating additional gene‐environment interactions and exploring how combined genetic and environmental factors influence the risk of spina bifida. Advanced genomic techniques such as whole‐exome and whole‐genome sequencing are promising tools for identifying novel genetic variants involved in these interactions, potentially leading to personalized prevention and treatment approaches.

### 4.3 Genetic alterations in the Wnt signaling pathway

The Wnt signaling pathway is a complex network of proteins that plays a role in key processes of embryonic development, cell differentiation, and tissue homeostasis.^134^ This pathway consists of two axes, the canonical and non‐canonical pathways.^135^ The two pathways differ because the canonical pathway is considered *β*‐catenin dependent. The Planar Cell Polarity (PCP) pathway is one of the non‐canonical and non *β*‐catenin dependent Wnt signaling pathways. It coordinates the orientation of cells along a tissue plane, which is central to enabling morphogenetic processes. Several genes that are essential to the PCP pathway include *CELSR1*, *VANGL1*, *VANGL2*, and *PTK7*, among others. Variations or interruptions to the regular function of these may result in issues establishing and/or maintaining cell polarity and correct directional cell movement, which can play a role in causing NTDs. It has been shown in the literature that mutations in Cadherin EGF LAG Seven‐Pass G‐Type Receptor 1 (*CELSR1*) contribute to increased risk of spina bifida. In one study, the authors found two specific TG indels in *CELSR1* to be associated with myelomeningocele.^136^ Interestingly, they highlighted another study which found SNVs altering CELSR1 protein function less severely to be associated with craniorachischisis (a more severe NTD).^136^, ^137^ Based on the current understanding of NTDs being caused by a combination of multiple genetic and environmental factors, they theorize that one functional mutation in *CELSR1* is insufficient to produce an NTD phenotype, which may explain the difference in findings between these two studies. In addition to *CELSR1*, other key genes in this pathway have been associated with increased spina bifida risk.

Another study examined 172 different SNPs with mostly negative results but did find that 46% of the SNPs with the smallest *p*‐values were variants of the *PRICKLE2* gene. The researchers noted that the small sample size and false discovery rate among multiple SNP testing limited the power of the study, but asserted that the observation of small *p*‐values in the *PRICKLE2* gene meant that they could not rule out the potential association between the gene and spina bifida.^138^ Yet another study investigated the role of variants in *SCRIB*, another PCP pathway‐associated gene, in modifying the risk for spina bifida and other NTDs. It found that deleterious mutations of *SCRIB* were associated with spina bifida, but unclear through what exact mechanism. It has been suggested, however, that such mutations may affect the interaction between *SCRIB* and *VANGL2*, with SCRIB typically acting as a scaffolding protein and VANGL2 directly participating in the formation of regulatory signaling complexes.^136^, ^139^ In conclusion, the literature highlights the complex interplay between gene mutations within the PCP pathway and their contributions to different forms of NTDs. Mutations in *CELSR1* and other PCP genes can disrupt cell polarity and movement, crucial for proper neural tube closure. The specific mutations and their interactions with other genetic and environmental factors influence whether the outcome is spina bifida, craniorachischisis, or other NTD subtypes. This underscores the importance of examining both genetic interactions and the broader genetic context to comprehensively understand the etiology of NTDs.

### 4.4 Genetic variants in the oxidative stress pathway

Excessive oxidative stress has also been implicated as a mechanism for spina bifida. The production or retention of reactive oxygen species (ROS) can cause damage if not properly and sufficiently eradicated. The accumulation of such damage, if not repaired, can affect lipids, proteins, and nucleic acids, and thus lead to various pathologies including NTDs. Oxidative stress is tightly regulated during embryonic development, and this process is important for normal cellular function and signaling. Superoxide dismutases (SODs) serve as antioxidant enzymes that catalyze the dismutation of superoxide radicals into oxygen and hydrogen peroxide, which is then further broken down through other antioxidant mechanisms. The primary SOD enzymes include SOD1 (cytoplasmic), SOD2 (mitochondrial), and SOD3 (extracellular). Because they play a crucial role in protection from oxidative damage by neutralizing superoxide radicals, proper functioning of *SOD* genes is essential for normal development. Along these lines, investigation of SNPs within the *SOD1* and *SOD2* genes, yielded four SNPs in *SOD1* (rs202446, rs202447, rs4816405, and rs2070424) and one SNP in *SOD2* (rs5746105) that were significantly associated with myelomeningocele risk.^140^ The following antioxidant enzymes, including SOD, display lower than typical levels of activity or concentration in infants with myelomeningocele and/or their mothers: carbonic anhydrase (CA), erythrocyte free radical scavenging enzymes (FRSE) included catalase (CAT), superoxide dismutase (SOD), glutathione peroxidase (GPX) and glutathione‐S‐ transferase (GST).^70^ In addition to any independent effects, there is also an important mechanism of interplay between oxidative stress and glucose homeostasis; oxidative stress can impair glucose metabolism, which can further increase ROS production due to excessively high glucose levels. A study investigating genes in the combined glucose homeostasis and oxidative stress (GHOS) network found that 22 of 568 studied genes were possible NTD risk genes (*p* < 0.05).^130^

### 4.5 More genome‐wide association studies with the technological advances in genome science

Advances in genetic sequencing technologies have shown promise for the large‐scale discovery of genes associated with spina bifida risk. Within these new technologies are two methods of detecting structural variations (SVs): array‐based detection and sequencing‐based computational methods. Array‐based methods, although advantageous for high‐throughput analysis, only detect specific types of SVs, have lower sensitivity for smaller SVs, and have lower resolution for determining breakpoints than sequencing‐based methods; however, sequencing requires more time and is expensive.^141^, ^142^ For detecting broad ranges of SVs, such as on a population scale, sequencing‐based methods are necessary.

Next Generation Sequencing (NGS) allows for the identification of variants in new genes previously not implicated in the pathogenesis of the disease.^143^ The advances of NGS allow for the possibility of the prediction of NTD risk, information about NTD polygenicity, and further evaluation of genomic variations.^141^ Whole genome sequencing (WGS), a type of NGS technology, is an approach to sequencing the entire DNA sequence of an organism, and has been used to identify rare and novel variants in several genes, including the *RAD9B* gene.^144^ WES, another type of NGS technology, is a genomic technique that allows for a detection of rare or novel variants in new genes that can contribute to a risk of NTDs. WES enables the generation of genome data from small amounts of input DNA and low‐cost testing of a substantial number of cohorts.^145^ For instance, WES was performed on six trios and was able to evaluate all possible ways of the genetic transmission of spina bifida: *de novo* dominant variants, homozygous and compound heterozygous variants, and X‐linked recessive variants. WES was able to identify *de novo* variants that could contribute to the risk of spina bifida in the patients.^143^ In another experiment, WES was applied to 506 myelomeningocele patients and detected ultra‐rare deleterious variants in approximately 70% of the subjects.^146^

However, to obtain a complete and accurate record of variation from sequencing data from NGS, there are many requirements and challenges, including mapping reads to the reference genome.^145^, ^147^, ^148^ Due to high cost, each read must be aligned independently, generating a high risk of misaligned reads spanning indels. Additionally, the per‐base quality scores are often inaccurate and vary with sequencing technology, machine cycle, and sequence context. These two issues lead to false SNP discovery and genotyping, severely affecting projects involving multiple sequencing technologies. Another limitation of current sequencing methods is the challenge of separating true variation from machine artifacts due to the high rate and context‐specificity of sequencing errors. Current approaches include filtering SNP calls with outlying characteristics. However, developing such filters require parameterization for each new data set and must be highly specific or highly sensitive. Furthermore, all challenges must be viewed in the context of a proliferation of sequencing technology platforms and study designs.

Despite these limitations, before the application of NGS technologies, genetic variants could only be detected one gene at a time, introducing bias by current knowledge of candidate genes as opposed to genome‐wide technologies.^149^ The process involves the amplification of exons of the chosen gene by polymerase chain reactions (PCR) to identify SNPs.^150^ Then, the PCR products are sequenced using two main technologies of Sanger Sequencing or the Prism Bigdye Terminator Kit, confirmation of the detected variants reached through repeat of PCR and sequencing. The main limitation of these technologies is the cost‐prohibitive deep sequencing processes, such as Sanger sequencing.^151^, ^152^ While the time commitment and high cost limit the number of samples that can be examined, future research with new generation sequencing technology can open doors to larger‐scale sequencing and variant detection.

## 5 CONCLUSIONS

Spina bifida represents a multifactorial birth defect influenced by a complex interplay of genetic and environmental factors. Despite significant advances in understanding the role of folic acid in preventing NTDs, many cases persist due to other risk factors such as exposure to environmental pollutants, adverse effects of medications, maternal diabetes, and obesity. Genetic predispositions, particularly those affecting the folate‐homocysteine pathway, oxidative stress, and Wnt signaling pathways, further complicate the pathogenesis of spina bifida.

Future research in spina bifida should focus on several key areas to further elucidate the etiology and enhance prevention and treatment strategies. One critical direction is the comprehensive integration of gene‐environment interactions using advanced genomic techniques. Whole‐genome and whole‐exome sequencing can uncover novel genetic variants and their interactions with environmental factors, providing a more detailed understanding of the multifactorial nature of spina bifida. Large‐scale genome‐wide association studies (GWAS) are particularly promising in identifying susceptibility loci that could inform targeted prevention efforts.^153^, ^154^ Additionally, the development of precision medicine approaches tailored to individuals’ genetic profiles holds significant potential for improving outcomes. Personalized supplementation strategies, such as adjusting folic acid intake based on specific genetic variants, could optimize preventative measures for those at higher risk.^155^ Research should also explore the mechanistic pathways through which environmental pollutants contribute to spina bifida, including endocrine disruptors and neurotoxins. Understanding these pathways could lead to the development of targeted interventions to mitigate these risks during critical periods of fetal development. From a clinical perspective, advancing prenatal screening techniques to include genetic testing for known risk variants could improve early detection and intervention. Incorporating routine genetic screening into prenatal care can help identify at‐risk pregnancies. Finally, translating these research findings into public health policies is crucial. Strengthening regulations on environmental pollutants and ensuring widespread access to folate‐rich foods and supplements can significantly reduce the incidence of spina bifida. Collaborations between investigators, clinicians, and policymakers will be essential to study and implement prevention strategies effectively. The ongoing exploration of these multifaceted risk factors underscores the need for a multidisciplinary approach to effectively reduce the incidence and impact of this disease.

## AUTHOR CONTRIBUTIONS

ESW, TCH, YZ, MJL, DAH, LSS, and RRR conceived and designed the study. ESW, DAH, LZ, RQ, and CX screened and assessed literatures through the search protocol. ESW drafted, revised, and executed the search protocol. ESW and DAH designed and created the figures and tables. ESW, DAH, LZ, RQ, CX, OM, GS, WY, and CL wrote, commented and revised the early drafts of the manuscript. All authors reviewed, edited, and approved the final manuscript.

## CONFLICT OF INTEREST STATEMENT

TCH and RRR serve on the Editorial Board of *Pediatric Discovery*. Both were excluded from all editorial decision‐making related to the acceptance of this article for publication. All other co‐authors declare no conflict of interest.

## ETHICS STATEMENT

Not applicable.

## Supporting information

Table S1

## Data Availability

The data that supports the findings of this study are available in the supplementary material of this article.
