# Supplementary material for: Spina bifida as a multifactorial birth defect: Risk factors and genetic underpinnings
Source: Pediatr Discov. 2025 Jan 25;3(2):e2517. doi: 10.1002/pdi3.2517 (PMC12258106; doi:10.1002/pdi3.2517)
Supplement: Supplementary file 1 — Table S1 [file PDI3-3-e2517-s001.docx]

**Supplement Table 1. Literature Search Protocol**

**Performed by**: Ethan S. Wong
**Search last executed**: April 8, 2024

**Database: NCBI PubMed**

| **Set name** | **Terms** | **Results** |
| --- | --- | --- |
| **Filters** | **Full text, Humans, English. 2003 to 2023.** |  |
| 1. Spina bifida | “Spinal dysraphism”[TI] OR “Spina bifida cystica”[TI] OR “Spina bifida occulta”[TI] OR “meningocele”[TI] OR “meningomyelocele”[TI] OR “spina bifida”[TI] OR “spinal dysraphisms”[TI] OR “myelomeningocele”[TI] OR “meningomyeloceles”[TI] OR “myelomeningoceles”[TI] OR “meningoceles”[TI] OR “lipomyelomeningocele”[TI] OR “lipomyelomeningoceles”[TI] OR “lipomeningomyelocele”[TI] OR “myeloschisis”[TI] OR “myelocele”[TI] OR “myeloceles”[TI] OR “lipomeningocele”[TI] OR “dermal sinus tract”[TI] OR “dermal sinus tracts”[TI] OR “spina bifida aperta”[TI] OR “occult spinal dysraphism”[TI] OR “spinal dysraphia”[TI] OR “closed spinal dysraphism”[TI] OR “open spinal dysraphism”[TI] OR “closed spinal dysraphisms”[TI] OR “open spinal dysraphisms”[TI] OR “spinal dysraphism”[MeSH Major Topic] | 3,917 |
| 1. Genetics | “Genes”[TIAB] OR “gene”[TIAB] OR “genetic”[TIAB] OR “genetics”[TIAB] OR “genome”[TIAB] OR “genomic”[TIAB] OR “genomics”[TIAB] OR “genomes”[TIAB] or “genetics”[MeSH] OR “genes”[MeSH] | 1,371,883 |
| 1. Risk Factors | “Causality”[TIAB] OR “precipitating factors”[TIAB] OR “protective factors”[TIAB] OR “risk factors”[TIAB] OR “etiology”[TIAB] OR “etiologies”[TIAB] OR “multifactorial causality”[TIAB] OR “multifactorial causalities”[TIAB] OR “causation”[TIAB] OR “multiple causation”[TIAB] OR “multiple causations”[TIAB] OR “causations”[TIAB] OR “causality”[MeSH] OR “risk factors”[MeSH] | 954,319 |
|  | 1 AND 2 | 228 |
|  | 1 AND 3 | 369 |
|  | 1 AND 2 AND 3 | 89 |

**Database: Embase via Elsevier**

|  | **Terms** | **Results** |
| --- | --- | --- |
| Filters for all searches | ([embase]/lim OR [preprint]/lim) NOT ([embase]/lim AND [medline]/lim) NOT ([medline]/lim) AND (2003:py OR 2004:py OR 2005:py OR 2006:py OR 2007:py OR 2008:py OR 2009:py OR 2010:py OR 2011:py OR 2012:py OR 2013:py OR 2014:py OR 2015:py OR 2016:py OR 2017:py OR 2018:py OR 2019:py OR 2020:py OR 2021:py OR 2022:py OR 2023:py) AND (‘article’/it) |  |
| 1. Spina bifida | “Spinal dysraphism”:ti OR “Spina bifida cystica”:ti OR “Spina bifida occulta”:ti OR “meningocele”:ti OR “meningomyelocele”:ti OR “spina bifida”:ti OR “spinal dysraphisms”:ti OR “myelomeningocele”:ti OR “meningomyeloceles”:ti OR “myelomeningoceles”:ti OR “meningoceles”:ti OR “lipomyelomeningocele”:ti OR “lipomyelomeningoceles”:ti OR “lipomeningomyelocele”:ti OR “myeloschisis”:ti OR “myelocele”:ti OR “myeloceles”:ti OR “lipomeningocele”:ti OR “dermal sinus tract”:ti OR “dermal sinus tracts”:ti OR “spina bifida aperta”:ti OR “occult spinal dysraphism”:ti OR “spinal dysraphia”:ti OR “closed spinal dysraphism”:ti OR “open spinal dysraphism”:ti OR “closed spinal dysraphisms”:ti OR “open spinal dysraphisms”:ti OR “spinal dysraphism”/exp | 1,792 |
| 1. Genetics | “Genes”:ti,ab OR “gene”:ti,ab OR “genetic”:ti,ab OR “genetics”:ti,ab OR “genome”:ti,ab OR “genomic”:ti,ab OR “genomics”:ti,ab OR “genomes”:ti,ab OR “gene”/exp OR “genetics”/exp | 351,010 |
| 1. Risk Factors | “Causality”:ti,ab OR “precipitating factors”:ti,ab OR “protective factors”:ti,ab OR “risk factors”:ti,ab OR “etiology”:ti,ab OR “etiologies”:ti,ab OR “multifactorial causality”:ti,ab OR “multifactorial causalities”:ti,ab OR “causation”:ti,ab OR “multiple causation”:ti,ab OR “multiple causations”:ti,ab OR “causations”:ti,ab OR “risk factor”/exp OR “etiology”/exp OR “causality”/exp | 369,351 |
|  | 1 AND 2 | 165 |
|  | 1 AND 3 | 226 |
|  | 1 AND 2 AND 3 | 49 |

**Database: Cochrane Library**

| **Set name** | **Terms** | **Results** |
| --- | --- | --- |
| **Filters** | **2003-2023** |  |
| 1. Spina bifida (Record title) | (“Spinal dysraphism” OR “Spina bifida cystica” OR “Spina bifida occulta” OR “meningocele” OR “meningomyelocele” OR “spina bifida” OR “spinal dysraphisms” OR “myelomeningocele” OR “meningomyeloceles” OR “myelomeningoceles” OR “meningoceles” OR “lipomyelomeningocele” OR “lipomyelomeningoceles” OR “lipomeningomyelocele” OR “myeloschisis” OR “myelocele” OR “myeloceles” OR “lipomeningocele” OR “dermal sinus tract” OR “dermal sinus tracts” OR “spina bifida aperta” OR “occult spinal dysraphism” OR “spinal dysraphia” OR “closed spinal dysraphism” OR “open spinal dysraphism” OR “closed spinal dysraphisms” OR “open spinal dysraphisms”) | 188 |
| 1. Genetics (Title, abstract, keyword) | (“Genes” OR “gene” OR “genetic” OR “genetics” OR “genome” OR “genomic” OR “genomics” OR “genomes”) | 81,433 |
| 1. Risk Factors (Title, abstract, keyword) | (“Causality” OR “precipitating factors” OR “protective factors” OR “risk factors” OR “etiology” OR “etiologies” OR “multifactorial causality” OR “multifactorial causalities” OR “causation” OR “multiple causation” OR “multiple causations” OR “causations”) | 201,610 |
|  | 1 AND 2 | 6 |
|  | 1 AND 3 | 44 |
|  | 1 AND 2 AND 3 | 4 |
